# Improving hydropower choices via an online and open access tool

**DOI:** 10.1371/journal.pone.0179393

**Published:** 2017-06-26

**Authors:** Thais Vilela, John Reid

**Affiliations:** Conservation Strategy Fund, Washington DC, United States of America; University of Vermont, UNITED STATES

## Abstract

This paper describes and validates the HydroCalculator Tool developed by Conservation Strategy Fund. The HydroCalculator Tool allows researchers, policy-makers and citizens to easily assess hydropower feasibility, by calculating traditional financial indicators, such as the levelized cost of energy, as well as greenhouse gas emissions and the economic net present value including emissions costs. Currently, people other than project developers have limited or no access to such information, which stifles informed public debate on electric energy options. Within this context, the use of the HydroCalculator Tool may contribute to the debate, by facilitating access to information. To validate the tool’s greenhouse gas calculations, we replicate two peer-reviewed articles that estimate greenhouse gas emissions from different hydropower plants in the Amazon basin. The estimates calculated by the HydroCalculator Tool are similar to the ones found in both peer-reviewed articles. The results show that hydropower plants can lead to greenhouse gas emissions and that, in some cases, these emissions can be larger than those of alternative energy sources producing the same amount of electricity.

## Introduction

Within the energy sector, one of the greatest challenges is to increase electricity supply in an economically feasible way while decreasing greenhouse gas emissions. Although clean and renewable sources, such as wind and solar, are becoming increasingly cost competitive, many countries, especially developing countries, are investing in large-scale hydropower plants to simultaneously meet growing demand for electricity and greenhouse gas emission targets. Studies such as Fearnside (2005) [1] and Faria et al. (2015) [2] show, however, that investing in hydropower plants to curb greenhouse gas emissions may not be the best approach as some hydropower plants may pollute as much as thermal power plants. Further, many hydro plants are not subjected to sufficient independent economic review and are selected despite unfavorable economic indicators.

Given this context, the non-governmental organization Conservation Strategy Fund developed the online HydroCalculator Tool (HCT) to facilitate the evaluation of hydropower plant projects. The HCT uses information provided by the user to calculate the environmental and social impact of these projects, as well as traditional measures of economic performance. Usually, large consultancy firms are tasked with evaluating hydropower projects. However, these evaluations are often not transparent, because firms do not make their methodology or the results publicly available. The HCT’s goals are to offer transparency and to improve decision making by automating calculation of traditional and non-traditional quantitative indicators. This allow a broad group of citizens, researchers and policy makers, to foresee and monitor the economic and environmental consequences of hydropower projects.

In this paper, we contribute to the ongoing discussion on hydropower plant emissions by reviewing the academic literature and validating the HCT, which, apart from including externalities, can be used by anyone. To validate HCT, we replicate the papers Abril et al. (2005) [3] and Faria et al. (2015) [2]. Specifically, we calculate the feasibility of several hydropower projects in the Amazon basin, by estimating traditional indicators, such as the levelized cost of energy (LCOE) and net present value (NPV), as well as greenhouse gas emissions and the NPV including greenhouse gas costs.

## Greenhouse gas emissions from hydropower plants

The current debate on hydropower plant emissions and on the role of hydroelectricity in the fight against climate change is relatively unknown outside the scientific community [4]. The first publication on this topic appeared in the early 1990s [5], but only now has the debate intensified. New findings on greenhouse gas emissions from tropical reservoirs, especially from Brazil, have sparked the debate [6–8].

The Intergovernmental Panel on Climate Change (IPCC) Annex III: Technology-specific cost and performance parameters [9] shows that greenhouse gas emissions from hydropower plants vary substantially, between 1 and 2200 g CO_2_eq/KWh. But, despite this variation, the distribution is not symmetric; on the contrary, it is positively skewed, with the median value equal to 24 g CO_2_eq/KWh. By comparison, the median values for natural gas, and coal are, respectively, 490, and 820 g CO_2_eq/KWh. These relatively low greenhouse gas emissions associated with hydropower plants on IPCC are, according to Fearnside (2015) [10], due to the use of studies mainly from temperate and boreal locations. Studies such as Barros et al. (2011) [11] provide evidence to this claim. These relatively low estimates underlie assertions as that made by Malovic et al. (2015) [12] that most hydropower projects generate sufficient electricity to more than offset the greenhouse gases that would otherwise have been produced by burning fossil fuels.

The controversy regarding hydropower greenhouse gas emissions may be explained by the amount of uncertainty related to emissions calculations, especially methane (CH_4_). Even the IPCC guidelines for reporting greenhouse gas emissions from hydropower appear to be limited. According to its report [13], “current measurements of CH_4_ fluxes from flooded land are not sufficiently comprehensive to support the development of accurate default emission factors.” Recent studies, however, have shown the significance of potent CH_4_ emissions from reservoirs and have contended that while there may be no accepted default measurement methodology, overlooking methane may seriously understate hydropower’s greenhouse gas (GHG) impacts [10]. Besides, contrary to the largely accepted standpoint, CH_4_ emissions from temperate reservoirs can be even higher than those from tropical reservoirs [14].

**Fig 1:**
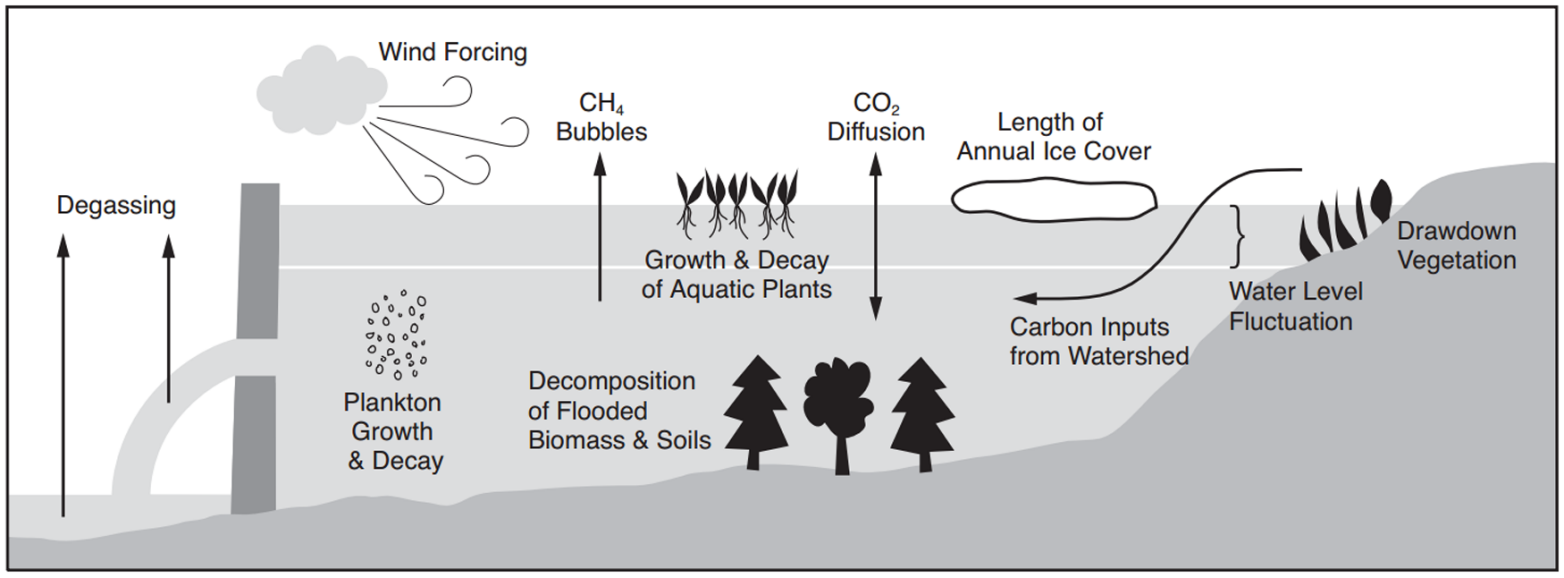
Major factors influencing reservoir GHG emissions.

Fig 1 shows some key factors affecting greenhouse gas emissions from reservoir [15]. Greenhouse gas emissions from the decomposition of flooded biomass and soils is the main source of hydropower emissions typically studied. This is due to the fact that the release of greenhouse gases due to biomass decomposition is the largest direct source [16]. Emissions from hydropower plants should also consider emissions from turbines and spillways, as pointed out in Fearnside (2016) [17]. But the lack of a common methodology, as well as the greater uncertainty associated with emissions from these sources, results in their exclusion from greenhouse gas calculations.

Another policy-relevant issue in the evaluation of hydropower projects is the time horizon used to standardize emissions for different greenhouse gases. To estimate the global warming impact of hydropower emissions, non-CO_2_ gases—in this case, CH_4_ –are converted to CO_2_-equivalent, by using the Global Warming Potential (GWP) determined by the IPCC. The GWP measures the relative radiative effect of a given substance compared to another, integrated over a chosen time horizon, which IPCC assumes to be or 20 or 100 years. To date, the 100-year time horizon has been most frequently used to convert the impact of CH_4_ emissions to CO_2_-equivalent. It is worth mentioning that IPCC offers no guidance regarding which time horizon to select and, according to Fearnside (2015) [10], this choice is critical. As the time horizon grows, the importance of CH_4_ relative to CO_2_ declines. While CO_2_ emissions tend to last for long periods of time, CH_4_ emissions remain in the atmosphere for approximately twelve years [18]. Therefore, using a 100-year time horizon tends to show lower emissions from hydropower and hence from countries such as Brazil, which relies on hydropower to produce almost 70% of its electricity.

## The HydroCalculator Tool

HCT is written in PHP and is based on the Drupal platform. It is a free software used to perform analysis of the economic feasibility of hydropower projects, as well as calculating some simple—yet important—environmental and social indicators. The tool can be accessed from http://www.conservation-strategy.org/en/hydrocalculator-analyses.

### Data

Due to HCT’s interactivity, most project-level data are supplied by users, but some default values, such as the wholesale price of energy, existing energy mixes, capacity factors and the discount rate, are provided by the tool (though they can be overridden). For calculating the NPV and the Internal Rate of Return (IRR), HCT uses the following data: flooded area, carbon density, construction cost, operation and maintenance costs, installed capacity, used capacity, wholesale price of energy, and, in the case of NPV, discount rate. Most of the data needed are project-specific, but for those data that are not easily obtained and that are more associated with the country’s condition, such as the project’s discount rate, HCT has default data for several countries in the world.

To calculate greenhouse gas emissions, HCT uses data from two sources: the IPCC Annex III: Technology-specific cost and performance parameters [9] and the Data Shift Portal [19]. From IPCC, HCT gets information on emission factors and on Global Warming Potential to convert CH_4_ into CO_2_-equivalent. From the Data Shift Portal, HCT gets, for each of the 44 countries in the database, data on electricity generation by energy source.

Ideally, to increase HCT’s precision, we would like to have data on CO_2_ and CH_4_ emissions throughout the analyzed period, but these data are not easily available for all existing hydro projects. Besides, this information would not be available for projects that are still in the planning stage. Therefore, some simplifications are needed. To estimate greenhouse gas emissions from hydropower reservoirs, HCT relies on a model based on carbon stock. This model allows the estimation of greenhouse gas emissions for both existing and future hydro projects, using available data. As an added benefit, this procedure makes the results for different projects—potentially in different countries—more comparable.

Finally, HCT assumes a value of 5.00 USD per ton of CO_2_eq. This price allows HCT to calculate the environmental cost resulting from the project’s greenhouse gas emissions or avoidance, as the case may be. This economic value, which represents the project’s climate externality, is subtracted from the traditional NPV. The resulting number is the NPV including greenhouse gas emission costs. While some estimates of the social cost of GHG emissions are more than an order of magnitude greater than this figure (from 14 USD per ton of CO_2_ to a value as high as 138 USD per ton of CO_2_ [20], the number we use more closely approximates the current market value of avoided carbon emissions and is a conservative estimate of the value of emissions reductions to the country achieving them [21].

### Methodology

#### Net present value and Internal Rate of Return

To calculate the economic feasibility of a hydro project, HCT estimates the NPV using the following formula:

$$\text{NPV}=\Sigma_{\text{t}=0}^{49}\frac{\left({\text{benefit}}_{\text{t}}-{\text{cost}}_{\text{t}}\right)}{{\left(1+\text{i}\right)}^{\text{t}}}$$ (1)

where i represents the discount rate and t the year—HCT assumes a time horizon equal to 50 years. The construction cost is divided equally across the estimated construction time period. Once operation starts, HCT calculates revenues as the wholesale price at the plant times annual production, which is the product of installed capacity and the average amount of capacity used, multiplied by the 8,760 hours in the year. For the annual operation and maintenance costs, HCT assumes that these costs are equal to four percent of capital costs. Finally, the choice of the discount rate is not trivial. HCT provides a default discount rate equal to 10 percent since this is the standard value used in development bank analyses of dam projects in developing countries. Nevertheless, because of the sensitivity of the NPV to this variable, users are encouraged to try different discount rates A high rate reduces the net present value of hydro projects, eliminating some dams from consideration before even considering environmental impacts.

The Internal Rate of Return (IRR), also used to measure the profitability of potential investments, is the discount rate that makes the NPV equal to zero. The IRR calculations rely on the same formula that NPV does:

$$0=\Sigma_{\text{t}=0}^{49}\frac{\left({\text{benefit}}_{\text{t}}-{\text{cost}}_{\text{t}}\right)}{{\left(1+\text{IRR}\right)}^{\text{t}}}$$ (2)

#### Levelized cost of energy

As with the NPV and IRR, the simplified LCOE is also computed for assessing the financial feasibility of hydro projects. The LCOE is interpreted as the minimum energy price at which energy must be sold for the hydropower project to break even. It represents the cost per megawatt-hour of building and operating, in our case, a hydropower plant over the life-cycle of the project, 50 years. To calculate the LCOE, we solve Eq (1) for the wholesale price of energy assuming that the NPV is zero. In this case, we have:

$$0=\sum_{\text{t}=0}^{49}\frac{\left({\text{P}}_{\text{t}}\cdot {\text{Q}}_{\text{t}}-{\text{C}}_{\text{t}}\right)}{{\left(1+\text{i}\right)}^{\text{t}}}\Rightarrow 0=\sum_{\text{t}=0}^{49}\frac{P_{t}\cdot Q_{t}}{{\left(1+\text{i}\right)}^{\text{t}}}-\sum_{\text{t}=0}^{49}\frac{C_{t}}{{\left(1+\text{i}\right)}^{\text{t}}}\Rightarrow \sum_{\text{t}=0}^{49}\frac{P_{t}\cdot Q_{t}}{{\left(1+\text{i}\right)}^{\text{t}}}=\sum_{\text{t}=0}^{49}\frac{C_{t}}{{\left(1+\text{i}\right)}^{\text{t}}}$$

In this simplify version, the price of energy does not change throughout the study-period, i.e., *P*_*t*_ = *P* ∀*t*. Thus, the final formula is:

$$\text{LCOE}=\;\frac{\sum_{\text{t}=0}^{49}\frac{{\text{C}}_{\text{t}}}{{\left(1+\text{i}\right)}^{\text{t}}}}{\sum_{\text{t}=0}^{49}\frac{{\text{Q}}_{\text{t}}}{{\left(1+\text{i}\right)}^{\text{t}}}}$$ (3)

where C_t_ is the total cost of the project in year t (investment, operation and maintenance costs), and Q_t_ is the amount of electricity produced by the hydropower plant in year t.

#### Net present value including greenhouse gas emission

Greenhouse gas emissions are estimated in two steps. First, we calculate unit CO_2_-equivalent emissions from each energy source used to produce electricity in each existing country in the HCT’s database. Second, we calculate CO_2_-equivalent emissions from the hydro project being evaluated. The two estimates are then combined to obtain the net greenhouse gas emissions of the project. More specifically, the net greenhouse gas emissions are calculated as the dam’s emissions minus the average emission required to generate the same amount of energy using the country’s current electricity generation matrix.

Emissions from the electricity generation matrix are calculated by multiplying the amount of electricity generated by each source in the matrix by the corresponding greenhouse gas emission factor, which is given by IPCC (2011) [22]. Then, to obtain the amount of emissions per megawatt-hour, for each country, we divide the total amount of electricity produced by the total amount of greenhouse gas emitted.

To estimate greenhouse gas emissions from hydropower reservoirs, we use the Biome Carbon Loss (BCL) model from Lima et al. (2007) [23]. Based on the initial carbon stock, this model calculates the carbon stock in the reservoir in each year, using the following formula:

$${\text{Carbon}}_{\text{t}}\;={\text{ Carbon}}_{0}\;\cdot \;\left(\frac{\text{exp}\left(\;-0.3\;\cdot \text{ t }\right)}{5}\;+\;\frac{\text{exp}\left(\;-0.03\;\cdot \text{ t }\right)}{3}\;+\;\frac{1}{2}\;\right)$$ (4)

where Carbon_t_ is the amount of carbon (by assumption, comprised solely of CO_2_ and CH_4_) in tons in year t; Carbon_0_ is the initial amount of carbon in tons; and t is the time in years. The initial amount of carbon is the carbon content of the vegetation existing in the area that will be flooded, and the time horizon is the life cycle of the hydro project. In each year, the difference between the initial and the final amount of carbon stock is equal to carbon emissions, as we assume that all the difference goes to the atmosphere.

To estimate CO_2_-equivalent, we go one step further and calculate the proportion of carbon emitted as CO_2_ and CH_4_. Lima et al. (2007) [23] show the proportion of CO_2_ and CH_4_ on carbon emissions for different hydropower plants in the Amazon Basin. Based on these estimates, we calculate the average proportion for each one of those greenhouse gases. We find that CO_2_ emissions are, on average, 73 percent of carbon emissions and CH_4_, 27 percent. Certainly, these values change from reservoir to reservoir, but to build a model capable of being used in different situations by non-specialists, we opt for using the average shares.

To convert carbon into CO_2_ and CH_4_, HCT multiplies the amount of carbon by 44/12 and by 16/12 to obtain CO_2_ and CH_4_ respectively. Then, to convert CH_4_ into CO_2_-equivalent, HCT uses the GWP available in the IPCC’s Fifth Assessment Report, published in 2013. As mentioned before, IPCC presents two values, depending on the time horizon. Over a lifespan of 20 years, IPCC shows that 1 ton of CH_4_ has the same impact on global warming as 84 tons of CO_2_. On the other hand, the importance of CH_4_ declines relative to CO_2_ over time. So, when taking into account a lifespan of 100 years, the impact of 1 ton of CH_4_ is the same as 28 tons of CO_2_.

Until now, the GWP that has been most frequently used in the literature is the one consistent with a 100-year time horizon. But, as highlighted in the IPCC, “there is no scientific argument for selecting 100 years compared with other choices” and “the choice of time horizon is a value judgement since it depends on the relative weight assigned to effects at different time” [24]. Based on the literature and adopting a conservative position, HCT uses a GWP equal to 28 to convert the impact of CH_4_ emissions into CO_2_-equivalents. If the user wants to know the amount of CO_2_-equivalent emitted by the hydro project using a different GWP, then we recommend the following calculation:

$$\;\tilde{{\text{CO}}_{2}\text{eq}}=\;0.27\;\cdot {\text{ CO}}_{2}\text{eq }\cdot \text{ GWP }+0.73\;\cdot {\text{ CO}}_{2}\text{eq}$$ (5)

where $\tilde{{\text{CO}}_{2}\text{eq}}$ is the new value for CO2-equivalent emissions, CO_2_eq is HCT’s output and GWP is the Global Warming Potential value that the user believes is more appropriate for methane.

Finally, to obtain an estimate on how much the hydro project avoids—or adds—in terms of greenhouse gas emissions compared to an equivalent amount of electricity generated from alternative sources, HCT subtracts the amount of greenhouse gas emissions produced for every MWh of electricity generated from the—greenhouse gas emission resulting from the hydro project. This relative measure of emission—called net greenhouse gas emissions—shows the environmental impact of adding a hydropower into the country’s electricity generation mix.

#### Limitations of the HydroCalculator Tool

Measuring GHG emissions from hydropower plants is a complex task. It involves, as mentioned before, the estimation of greenhouse gas from not only the decomposition of flooded biomass and soils, but also turbines and spillways. In this sense, the Biome Carbon Loss model is a simple model. It considers emissions from the decomposition of organic matter, but does not account for GHG emissions from turbines and spillways. There is no single model—or a common methodology—that accounts for the latter and that could be generalized and applied to specific hydropower projects. Because of the greater uncertainty associated with emissions from these sources, HCT excludes these sources from greenhouse gas calculations. As a result, the estimates on CO_2_-equivalent are likely underestimated.

Genereux et al. (2013) [25] also highlighted the importance of taking into account the role of deep groundwater when estimating carbon fluxes in watersheds or, in this case, reservoirs. The result found suggests that not knowing the groundwater flow, more specifically the amount of carbon transported by the flow beneath surface, would most likely lead to incorrect carbon measures. There is, however, a significant research gap—as mentioned by the authors—on this topic.

Further, the current iteration of the model also does not take into account the changes in the mix of alternative energy sources that may take place over the lifetime of the project. A shift to greater reliance on high-emission sources, such as coal, would increase the greenhouse benefits of a given hydro project, while the opposite would be true if a country’s mix of electricity sources shifted toward wind and solar over the long-term. Due to the difficulty in forecasting these changes for dozens of countries, the energy mixes are left static, but can be continually updated by the tool’s administrator.

Finally, the model assumes that all the existing biomass is flooded. In some instances, vegetation is cleared or burned from reservoir areas. This assumption may increase the projected emissions by introducing methane emissions, which are minimal in a burning scenario. A future version of the model may present users with the option of a clearing and burning rather than assuming that biomass is flooded.

These limitations make it clear that there is a tradeoff between accessibility to non-specialist users and a high degree of precision on the climate change consequences of a project. The HCT does not substitute for in-depth project evaluations and environmental impact assessment. However, even within the constraints of a simplified carbon model, the tool can contribute to the debate on hydro options, which, within a given country, will be similarly affected by error in projecting net carbon emissions. Further, the tool’s limitations have less impact on the reliability of its pure economic indicators.

## Results

In this section, we present two sets of results. First, we show the results regarding the validation exercise, in which we compare emissions from hydropower projects using HCT with emission estimations from two peer-reviewed papers. Second, to illustrate the broader applicability of HCT for assessing the feasibility of hydropower projects, we show some traditional quantitative indicators, such as the NPV and IIR.

Table 1 shows the estimates on carbon emissions from Abril et al. (2005) [3] and HCT. Although HCT’s output is total emission in CO_2_-equivalent units, the comparison is useful to validate the BCL model. The result found by HCT is very close to the value reported in Abril et al. (2005) [3] for the Petit-Saut reservoir in French Guiana.

**Table 1:** Estimates on carbon emission from Abril et al. (2005) [3] and HCT.

Original parameters	Abril et al. (2005)	HCT
Initial amount of carbon: 10 Million tons of carbon; Time horizon: 10 years	2.2 Million tons of carbon	2.4 Million tons of carbon

Table 2 shows the estimates on greenhouse gas emissions found in Faria et al. (2015) [2] and calculated by the HCT. Faria et al. (2015) [2] use two different approaches to predict the future amount of carbon of eighteen reservoirs in the Amazon Basin. In both approaches, a Monte Carlo simulation is used to estimate greenhouse gases that go into the atmosphere. The results obtained from the first approach, which is based on emissions from flooded carbon stock (flooded soils and foliage, plus cleared biomass), are used as a reference to compare with the results found by the HydroCalculator.

**Table 2:** Comparison between Faria et al. (2015) [2] and HCT estimates on greenhouse gas emissions.

Hydroelectric power plant	River	tg CO_2_eq in 100 years ([2]; GWP_CH4_ is 34)	tg CO_2_eq in 50 years (HCT; GWP_CH4_ is 28)
Belo Monte	Xingu	47 (24–82)	49.6
Bem Querer	Branco	74 (36–129)	43.0
Cachoeira do Caí	Jamanxim	74 (41–121)	65.7
Cachoeira do Caldeirão	Araguari	6 (3–10)	5.7
Cachoeira dos Patos	Jamanxim	18 (9–31)	12.7
Colíder	Teles Pires	23 (11–42)	10.1
Ferreira Gomes	Araguari	2 (1–3)	1.0
Jamanxim	Jamanxim	13 (7–22)	10.0
Jatobá	Tapajós	47 (24–82)	58.2
Jirau	Madeira	42 (22–70)	35.8
Marabá	Tocantins	113 (54–201)	70.6
Salto Augusto de Baixo	Juruena	14 (8–25)	9.7
Santo Antônio	Madeira	31 (16–52)	26.7
São Luís do Tapajós	Tapajós	72 (38–123)	86.2
São Manoel	Teles Pires	10 (5–17)	7.9
São Simão Alto	Juruena	44 (24–73)	10.9
Sinop	Teles Pires	49 (23–88)	19.2
Teles Pires	Teles Pires	21 (10–37)	12.1

The numbers in parenthesis correspond to the 95% confidence interval

To calculate the amount of CO_2_-equivalent emitted into the atmosphere, HCT uses the average carbon content, as well as the reservoir area, installed capacity and capacity factor, presented in Faria et al (2015) [2]. Although HCT uses a less sophisticated approach, the results found are similar, and, except for two out of 18 hydropower plants, the estimates are within the 95 percent confidence interval calculated in [2].

HCT is not able to perfectly match the results because it uses different assumptions. While Faria et al. (2015) [2] use a time horizon equal to 100 years, HCT assumes 50 years. This means that our results are likely to have a downward bias. Another difference regards the GWP. Although both estimations use the GWP associated with a 100-year time horizon, due to revisions on IPCC, the values are different. While Faria et al. (2015) [2] use a GWP equal to 34, HCT uses 28. This difference also causes our estimates to be downward bias.

Table 3 presents the results on economic evaluation for some of the hydropower plants in Table 2 using official data available on [26]. The results show that, from an economic perspective, Belo Monte and Jirau are economically feasible with an IRR equal to 10.6 and 10.4 percent respectively. Regarding the other Brazilian hydropower plants evaluated here, the present value of the costs exceeds the present value of the benefits and, therefore, they are not economically feasible, which is also reflected in the LCOE values higher than the wholesale price of energy.

**Table 3:** Economic evaluation for hydropower plants (50-year time horizon).

Hydropower plant	Construction time	Start of operation	Investments (Brazilian Real, R$)	Wholesale price of energy (R$/MWh)	Levelized cost of energy (R$/MWh)	NPV (Thousand Brazilian Real, R$)	NPV including net GHG emissions (Thousand Brazilian Real, R$)	IRR (%)
Belo Monte	4	2015	19,018,115,000	77.97	74.00	1,132	1,295	10.6
Colíder	3	2014	1,266,264,270	103.40	130.00	-324,309	-358,793	7.19
Jirau	3	2013	8,699,124,120	71.37	69.00	342,670	367,302	10.4
Santo Antônio	4	2012	9,495,381,160	78.87	83.00	-592,611	-541,405	9.34
Teles Pires	4	2015	3,328,545,560	58.35	61.00	-159,714	-132,097	9.49

Financial figures for each project are in the currency of the year when concession contracts were approved. The IRR provides a basis for feasibility comparison of projects in different years. Information on *construction time*, *operation year*, *investment* and *wholesale price of energy* are from the concession contracts available at ANEEL (Agência Nacional de Energia Elétrica). The *wholesale price of energy* corresponds to the winner bid in the auction for the concession on the hydropower plant.

With the exception of the Colíder project, including net greenhouse gas emission costs increases the NPV, meaning that the net greenhouse gas emission costs are negative. For most hydropower projects analyzed here, emissions from the existing energy generation matrix are higher than the gross greenhouse gas emissions calculated for the hydro project. This is likely to happen in situations in which the alternative source of electricity is heavily dependent on burning fossil fuels and when the amount of biomass flooded by the hydro project is small relative to its energy output.

The results presented in Table 3 corroborate the common-sense findings in Ledec and Quintero (2003) [27] that dams vary in their efficiency and impact and that large hydroelectric dams can be either good or bad dams. However, in developing countries, like Brazil, using data based on concession contracts, as done in this paper, can have drawbacks. Because of conflicting priorities and weak institutions, the conditions asserted on the contracts may change through time. For example, the actual cost of the Belo Monte project is, approximately, 52 percent more than anticipated, changing our conclusion about its feasibility. Therefore, one must take caution when choosing the data and analyzing the results.

To expand our analysis of the impact of considering greenhouse gases on overall project feasibility beyond Brazilian hydropower projects, Table 4 presents the results for other hydropower projects in the Amazon basin. Data are from [28–32]. For all hydropower plants analyzed, the NPV is negative. When including CO_2_- equivalent emission costs, the results, in terms of project feasibility, do not change; when valued at the 5 USD per ton of CO_2_eq, as we do here, the GHG costs are not large enough to impact hydro project feasibility. For the Rositas project in Bolivia, CO_2_eq emission costs reduce the NPV, meaning that, from an environmental standpoint, the Rositas project represents a loss in terms of greenhouse gas emissions when compared to the alternative energy sources producing the same amount of electricity in Bolivia. For all other hydropower plants shown in Table 4, the savings in terms of greenhouse gas emissions is not sufficient to make the NPV greater than zero and, therefore, to make the projects economically feasible.

**Table 4:** Project evaluation for some hydropower plants in the Amazon basin (50-year time horizon).

Country	Hydropower plant	Construction time	Investments (nominal thousand USD)	Wholesale price of energy(USD/MWh)	Levelized cost of energy(USD/MWh)	NPV(nominal thousand USD)	IIR (%)	NPV including net GHG emission (nominal thousand USD)	Gross CO_2_e (nominal thousand USD)
Peru	Inambari	5	5,194	56.00	61.00	-493,223	8.98%	-443,439	33,772
Colombia	Sogamoso	5	1,740	40.00	57.00	-564,175	6.24%	-555,254	2,003
Ecuador	Cardenillo	5	1,135	40.9	55.00	-317,390	6.81%	-282,002	14,857
Bolivia	Rositas	7	1,000	40.00	101.00	-597,804	1.08%	-626,295	30,050

The choice of the hydropower plants evaluated in this table was arbitrary.

## Conclusions

The role of hydroelectric dams in fighting climate change continues to be a matter of debate, one that is likely to intensify as pressure grows to meet expanding electricity demand and rein in greenhouse gas emissions. In this context, it is critical that citizens, civil society groups, lenders, companies and potentially dam-affected people all have access to tools and information to understand the tradeoffs involved. Within this context, by allowing anyone to easily evaluate hydropower projects, HCT contributes to increase transparency of the energy and environmental policy debates, as well as of the decision-making process, by allowing specific recommendations.

Contrary to common perceptions, hydropower plants are not blame-free in terms of greenhouse gases emissions, especially in places such as the Amazon basin, with both abundant hydro resources and high concentrations of terrestrial carbon stocks. We found that considering GHG made some projects more economically beneficial and others less so. At the low carbon price of 5 USD per ton of CO2-equivalent, however, it did not change the overall conclusion about any project’s feasibility.

These findings are based on a small sample of projects selected purely for the purpose of validating the soundness of the HCT’s calculations and demonstrating the integration of GHG costs in its estimates. However, the information presented here points to the need for early, holistic evaluation of project feasibility, taking into account not only traditional project measures, but also environmental and social indicators. Within this context, the NGO Conservation Strategy Fund built the HydroCalculator Tool. Despite its simplicity, it contributes to the discussion by providing an easy way for ordinary users to calculate dams’ economic feasibility, including environmental costs, which are rarely taken into account quantitatively in the context of policymaking and hydropower projects.

Finally, to keep up to date with the latest findings in the hydropower GHG emission literature, CSF is continuously improving the HydroCalculator Tool. One of the main refinements, for example, is to incorporate emission from degassing (as the water is released from turbines) and downstream emissions. Taking into account both emissions will likely increase our estimates. Another important refinement regards the social carbon price. Currently, HCT uses a conservative flat value, but studies, such as Luckow et al. (2015) [33] and Kossoy et al. (2015) [21], show that the carbon price will likely follow an upward trend in the next forty years. If this is the case, then the higher carbon price will, holding everything else constant, decrease the project’s NPV, changing, therefore, its feasibility.

## Supporting information

S1 Supporting Information The supplementary material describes the required input data, presents the assumptions and input default values, and shows the output interface. (DOCX)
