# Supplementary material for: Improving hydropower choices via an online and open access tool
Source: PLoS One. 2017 Jun 26;12(6):e0179393. doi: 10.1371/journal.pone.0179393 (PMC5484472; doi:10.1371/journal.pone.0179393)
Supplement: S1 Supporting Information — (DOCX) [file pone.0179393.s001.docx]

**Improving Hydropower Choices via an Online and Open Access Tool**

**T Vilela^[[1]](#footnote-1)^ and J Reid**

Conservation Strategy Fund. 1636 R St. NW, Suite 3. Washington, DC 20009. United States.

E-mail: thais@conservation-strategy.org

**Supplementary Material**

This supplementary document is organized as follows. Section 1 presents an acronym dictionary. Section 2 describes the required input data. Section 3 presents the assumptions and the input default values used by the Conservation Strategy Fund’s HydroCalculator Tool (HCT). And Section 4 shows the output interface.

1. **Acronym Dictionary**

CH4 – Methane

GHG – Greenhouse gas

CO2 – Carbon dioxide

CO2-equivalent – Carbon dioxide equivalent

CSF – Conservation Strategy Fund

GWP - Global Warming Potential

HCT – HydroCalculator Tool

IPCC - Intergovernmental Panel on Climate Change

IRR – Internal rate of return

LCOE - Levelized cost of energy

MWh – Megawatt hours

NPV - Net present value

Tg - Teragrams

USD – United States dollar

1. **Required Input Data**

This section describes the data needed, and what should be provided by the user, in each step of the HCT tool, which can be accessed from http://conservation-strategy.org/en/hydrocalculator-analyses

**Basic Information**

In this step, the user must provide the following information:

- The name of the project;
- The status of the project (complete, construction scheduled, in operation, etc.); and
- The name of the country, city and river where the project is located. The tool helps the user pinpoint the location of the dam with the use of a Google map interface.


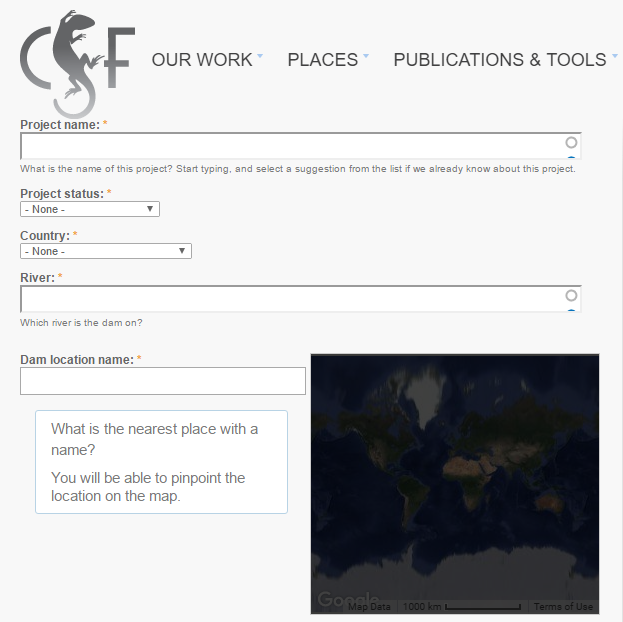


**Fig 1.** Basic information description

**Area and People**

The second step of the HCT requires accurate data on:

- The area flooded (in hectares); and
- The number of people displaced by the project.


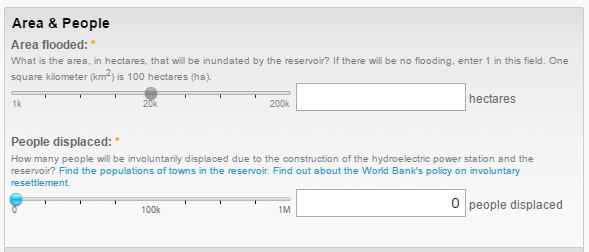


**Fig 2.** Area and people input data

**Vegetation and Carbon Density**

The type of vegetation in the area that will be flooded is assessed next. The HCT will automatically specify a vegetation type with its associated carbon density that corresponds to the area according to its built-in geographic database. But, the user may refine this information by:

- Specifying one or more types of vegetation from provided drop-down lists existing in the area that will be flooded. If there is more than one, the user must select the percentage of each type in this step.
- If known, specifying the carbon contents of the vegetation type. If none is specified, default values are used for each vegetation type.


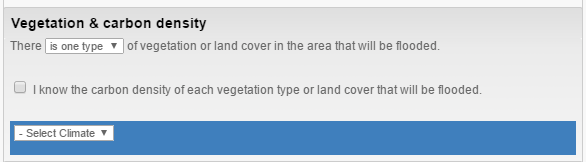


**Fig 3**. Vegetation and carbon density input data

**Installed Capacity and Construction**

In this step, the user provides information regarding:

- The construction time (in years), including the time taken to phase-in turbines after the construction is complete;
- The installed capacity (in MW); and
- The average capacity used (measured as a percentage of installed capacity).


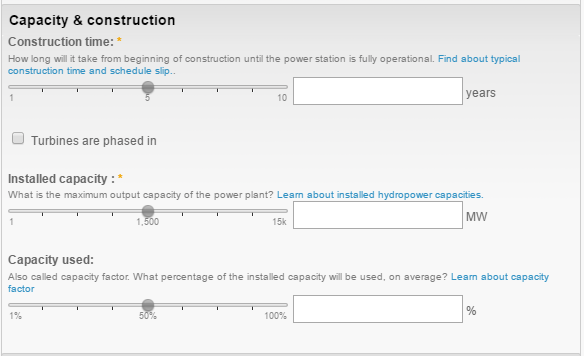


**Fig 4**. Capacity and construction input data

**Financial Values**

This is the final step. The user must enter the following information:

- The currency in which the calculation is being made;
- The construction and transmission costs of the project;
- The wholesale price of energy; and
- The economic discount rate.


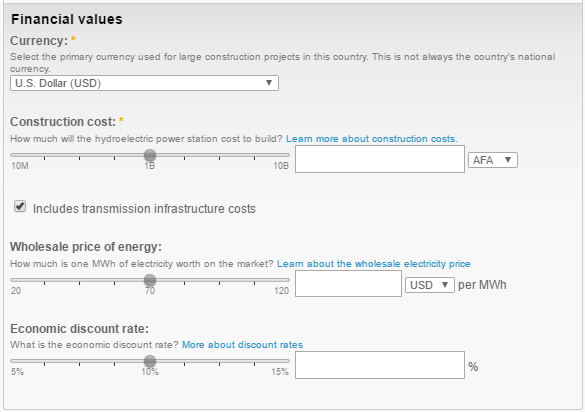


**Fig 5**. Financial values

1. **Assumptions and input default values**

**Assumptions**

HCT has three main assumptions:

- The total number of periods to calculate the NPV is 50 years. HCT assumes that after this time the impact of future cash flows on NPV is not significant;
- The operation and maintenance costs are 4 percent of construction costs, and the project begins operating in the first year after construction is completed; and
- The financial to economic conversion factors are equal to 1.

**Default values**

The HC tool has default values for five key variables:

- Wholesale price of energy (in US$);
- Discount rate (in %);
- Value per ton of CO2 equivalent emissions (in US$);
- Carbon content existing in each vegetation type; and
- Electricity’s CO2 equivalent emissions factor (in TCO2/MWh).

The user can specify input values for the first three variables and provide the carbon content of the area flooded for the fourth variable. If the user does not have this information, HCT uses its default for those input, according to the country and the ecosystem where the project will be carried out. The value of carbon emissions is fixed by the tool, though future versions may allow for this value to be varied by users. At the end of this document, we show the default values, as well as the methodology used to obtain these values, for each one of the variables.

**The wholesale price of energy**

To find the wholesale price of energy for each country, we use different sources, including articles, electricity generating company websites, and energy statistics databases.

**The discount rate**

For most countries, the real discount rate used is 12 percent per year. For some countries, such as the United States and Canada, however, the discount rate equals 6 percent. Besides the type of investment, the discount rate is also a function of the country risk premium. Table 1 shows the default discount rates for each country in the HCT.

Due to NPV’s sensitivity to the discount rate, CSF recommends that the user test different discount rates. In the section Data Source, we present several links to help the user better understand and decide which discount rates are most appropriate for their specific project.

**Table 1.** Wholesale energy prices and discount rates.

| Country | Wholesale energy Price | Discount rate |
| --- | --- | --- |
| Argentina | 50.00 USD | 12% |
| Bhutan | 50.00 USD | 12% |
| Bolivia | 40.00 USD | 12.80% |
| Brazil | 100.00 BRL | 10% |
| Burkina Faso | 50.00 USD | 12% |
| Burundi | 75.00 USD | 12% |
| Cambodia | 200,000.00 KHR | 12% |
| Canada | 50.00 CAD | 6% |
| Central African Republic | 50.00 USD | 12% |
| Chile | 65.40 USD | 12% |
| China | 45.00 USD | 12% |
| Colombia | 100,000.00 COP | 12% |
| Costa Rica | 25,000.00 CRC | 12% |
| Democratic Republic of Congo | 57.03 USD | 12% |
| Ecuador | 65.00 USD | 10% |
| Ethiopia | 50.00 USD | 12% |
| Ghana | 50.00 USD | 12% |
| Guatemala | 60.00 USD | 12% |
| Guinea | 126.57 USD | 12% |
| Guyana | 50.00 USD | 12% |
| Honduras | 50.00 USD | 12% |
| India | 80.00 USD | 12% |
| Kenya | 66.00 USD | 12% |
| Laos | 50.00 USD | 12% |
| Lesotho | 57.03 USD | 12% |
| Mali | 50.00 USD | 12% |
| Mexico | 50.00 USD | 12% |
| Mozambique | 57.03 USD | 12% |
| Nepal | 50.00 USD | 12% |
| Nicaragua | 50.00 USD | 12% |
| Pakistan | 50.00 USD | 12% |
| Panama | 50.00 USD | 12% |
| Peru | 157.00 PEN | 11% |
| Rwanda | 67.00 USD | 12% |
| Senegal | 130 EUR | 12% |
| Sudan | 70.00 USD | 12% |
| Tanzania | 57.03 USD | 12% |
| Thailand | 50.00 USD | 12% |
| Uganda | 70.00 USD | 12% |
| United States | 50.00 USD | 6% |
| Venezuela | 65.00 USD | 10% |
| Vietnam | 50.00 USD | 12% |
| Zambia | 57.03 USD | 12% |
| Zimbabwe | 57.03 USD | 12% |

**The price per ton of CO2**

For all countries, HCT assumes a value of 5.00 USD per ton of CO_2_-Equivalent. This price allows HC to calculate the environmental cost resulting from the project’s greenhouse gas emissions or avoidance, as the case may be. This economic value, which represents the project’s climate externality, is subtracted from the traditional NPV. The resulting number is the “green” NPV, which includes the economic costs/benefits of greenhouse gas changes.

**The carbon content in each vegetation type**

Table 2 shows the default data on carbon content per hectare used in the HCT tool along with the description of the vegetation in the area that will be flooded by the project. The characterization of the vegetation is done by the user using drop down lists that specify the climate, land cover, and other data. If there are multiple types of vegetation, then the user can enter in the HCT the percentage of each vegetation regarding the whole area that will be flooded by the dam.

Based on the carbon density and on the Biome Carbon Loss model, HCT calculates greenhouse gas emissions resulting from the hydro project. The methodology to calculate CO_2_-equivalent emission is presented in the paper.

**Table 2.** Carbon content by vegetation type

| Vegetation Type | Carbon cont. (t/ha) |
| --- | --- |
| Africa; Subtropical Humid Forest; Broadleaf/Needleleaf/Mixed Forest | 134.00 |
| Africa; Subtropical Humid Forest; Burnt, Natural Forest Mosaic | 67.00 |
| Africa; Subtropical Humid Forest; Forest, Cropland Mosaic; Non-Frontier | 15.00 |
| Africa; Subtropical Mountain Systems; Broadleaf/Needleleaf/Mixed Forest | 30.00 |
| Africa; Subtropical Mountain Systems; Burnt, Natural Forest Mosaic | 15.00 |
| Africa; Subtropical Shrub Cover | 43.00 |
| Africa; Tropical Dry Forest; Broadleaf/Needleleaf/Mixed Forest | 72.00 |
| Africa; Tropical Dry Forest; Burnt, Natural Forest Mosaic | 36.00 |
| Africa; Tropical Dry Forest; Forest, Cropland Mosaic; Non-Frontier | 36.00 |
| Africa; Tropical Moist Deciduous Forest; Broadleaf/Needleleaf/Mixed Forest | 152.00 |
| Africa; Tropical Moist Deciduous Forest; Burnt, Natural Forest Mosaic | 76.00 |
| Africa; Tropical Moist Deciduous Forest; Forest, Cropland Mosaic; Non-Frontier | 76.00 |
| Africa; Tropical Mountain Systems; Broadleaf/Needleleaf/Mixed Forest | 69.00 |
| Africa; Tropical Mountain Systems; Burnt, Natural Forest Mosaic | 34.50 |
| Africa; Tropical Mountain Systems; Forest, Cropland Mosaic; Non-Frontier | 34.50 |
| Africa; Tropical Rainforest; Broadleaf/Needleleaf/Mixed Forest | 200.00 |
| Africa; Tropical Rainforest; Burnt, Natural Forest Mosaic | 100.00 |
| Africa; Tropical Rainforest; Forest, Cropland Mosaic; Non-Frontier | 100.00 |
| Africa; Tropical Shrub Cover | 46.00 |
| Americas; Subtropical Dry Forest; Broadleaf/Needleleaf/Mixed Forest | 126.00 |
| Americas; Subtropical Dry Forest; Burnt, Natural Forest Mosaic | 63.00 |
| Americas; Subtropical Dry Forest; Forest, Cropland Mosaic; Non-Frontier | 63.00 |
| Americas; Subtropical Humid Forest; Broadleaf/Needleleaf/Mixed Forest | 128.00 |
| Americas; Subtropical Humid Forest; Burnt, Natural Forest Mosaic | 64.00 |
| Americas; Subtropical Humid Forest; Forest, Cropland Mosaic; Non-Frontier | 64.00 |
| Americas; Subtropical Mountain Systems; Broadleaf/Needleleaf/Mixed Forest | 87.00 |
| Americas; Subtropical Mountain Systems; Burnt, Natural Forest Mosaic | 43.50 |
| Americas; Subtropical Mountain Systems; Forest, Cropland Mosaic; Non-Frontier | 43.50 |
| Americas; Temperate Cont. Forest; Broadleaf/Needleleaf Forest; Frontie | 75.00 |
| Americas; Temperate Cont. Forest; Broadleaf/Needleleaf Forest; Non-Frontier | 36.00 |
| Americas; Temperate Cont. Forest; Burnt, Natural Forest Mosaic; Frontier | 38.50 |
| Americas; Temperate Cont. Forest; Burnt, Natural Forest Mosaic; Non-Frontier | 19.25 |
| Americas; Temperate Cont. Forest; Forest, Cropland Mosaic; Non-Frontier | 19.25 |
| Americas; Temperate Cont. Forest; Mixed Forest; Frontier | 77.00 |
| Americas; Temperate Cont. Forest; Mixed Forest; Non-Frontier | 38.50 |
| Americas; Temperate Mountain Systems; Broadleaf/Needleleaf/Mixed Forest; Frontier | 79.00 |
| Americas; Temperate Mountain Systems; Broadleaf/Needleleaf/Mixed Forest; Non-Frontier | 33.00 |
| Americas; Temperate Mountain Systems; Burnt, Natural Forest Mosaic; Frontier | 77.00 |
| Americas; Temperate Mountain Systems; Burnt, Natural Forest Mosaic; Non-Frontier | 33.50 |
| Americas; Temperate Mountain Systems; Forest, Cropland Mosaic; Non-Frontier | 16.75 |
| Americas; Temperate Oceanic Forest; Broadleaf Forest | 105.00 |
| Americas; Temperate Oceanic Forest; Burnt, Natural Forest Mosaic | 51.75 |
| Americas; Temperate Oceanic Forest; Mixed Forest | 103.50 |
| Americas; Tropical Dry Forest; Broadleaf/Needleleaf/Mixed Forest | 126.00 |
| Americas; Tropical Dry Forest; Burnt, Natural Forest Mosaic | 63.00 |
| Americas; Tropical Dry Forest; Forest, Cropland Mosaic | 63.00 |
| Americas; Tropical Moist Deciduous Forest; Broadleaf/Needleleaf/Mixed Forest | 128.00 |
| Americas; Tropical Moist Deciduous Forest; Burnt, Natural Forest Mosaic | 64.00 |
| Americas; Tropical Moist Deciduous Forest; Forest, Cropland Mosaic; Non-Frontier | 64.00 |
| Americas; Tropical Mountain Systems; Broadleaf/Needleleaf/Mixed Forest | 87.00 |
| Americas; Tropical Mountain Systems; Burnt, Natural Forest Mosaic | 43.50 |
| Americas; Tropical Mountain Systems; Forest, Cropland Mosaic; Non-Frontier | 43.50 |
| Americas; Tropical Rainforest; Broadleaf/Needleleaf/Mixed Forest | 193.00 |
| Americas; Tropical Rainforest; Burnt, Natural Forest Mosaic | 96.50 |
| Americas; Tropical Rainforest; Forest, Cropland Mosaic; Non-Frontier | 96.50 |
| Bare Areas | 1.00 |
| Cont Asia; Tropical Moist Deciduous Forest; Burnt, Natural Forest Mosaic | 52.50 |
| Cont. Asia; Subtropical Dry Forest; Broadleaf/Needleleaf/Mixed Forest | 78.00 |
| Cont. Asia; Subtropical Dry Forest; Burnt, Natural Forest Mosaic | 39.00 |
| Cont. Asia; Subtropical Dry Forest; Forest, Cropland Mosaic; Non-Frontier | 39.00 |
| Cont. Asia; Subtropical Humid Forest; Broadleaf/Needleleaf/Mixed Forest | 105.00 |
| Cont. Asia; Subtropical Humid Forest; Burnt, Natural Forest Mosaic | 52.50 |
| Cont. Asia; Subtropical Humid Forest; Forest, Cropland Mosaic; Non-Frontier | 52.50 |
| Cont. Asia; Subtropical Mountain Systems; Broadleaf/Needleleaf/Mixed Forest | 81.00 |
| Cont. Asia; Subtropical Shrub Cover | 37.00 |
| Cont. Asia; Temperate Cont. Forest; Broadleaf Forest; Frontier | 69.00 |
| Cont. Asia; Temperate Cont. Forest; Broadleaf/Needleleaf Forest; Non-Frontier | 14.00 |
| Cont. Asia; Temperate Cont. Forest; Burnt, Natural Forest Mosaic; Frontier | 35.50 |
| Cont. Asia; Temperate Cont. Forest; Burnt, Natural Forest Mosaic; Non-Frontier | 6.75 |
| Cont. Asia; Temperate Cont. Forest; Forest, Cropland Mosaic; Non-Frontier | 6.75 |
| Cont. Asia; Temperate Cont. Forest; Mixed Forest; Frontier | 71.00 |
| Cont. Asia; Temperate Cont. Forest; Needleleaf Forest; Frontier | 73.00 |
| Cont. Asia; Temperate Cont. Forest; Needleleaf Forest; Non-Frontier | 13.50 |
| Cont. Asia; Temperate Mountain Systems; Broadleaf Forest; Frontier | 75.00 |
| Cont. Asia; Temperate Mountain Systems; Broadleaf Forest; Non-Frontier | 58.00 |
| Cont. Asia; Temperate Mountain Systems; Burnt, Natural Forest Mosaic; Frontier | 38.50 |
| Cont. Asia; Temperate Mountain Systems; Burnt, Natural Forest Mosaic; Non-Frontier | 29.75 |
| Cont. Asia; Temperate Mountain Systems; Forest, Cropland Mosaic; Non-Frontier | 29.75 |
| Cont. Asia; Temperate Shrub Cover | 7.40 |
| Cont. Asia; Tropical Dry Forest; Broadleaf/Needleleaf/Mixed Forest | 78.00 |
| Cont. Asia; Tropical Dry Forest; Burnt, Natural Forest Mosaic | 39.00 |
| Cont. Asia; Tropical Dry Forest; Forest, Cropland Mosaic; Non-Frontier | 39.00 |
| Cont. Asia; Tropical Moist Deciduous Forest; Broadleaf/Needleleaf/Mixed Forest | 105.00 |
| Cont. Asia; Tropical Moist Deciduous Forest; Forest, Cropland Mosaic; Non-Frontier | 52.50 |
| Cont. Asia; Tropical Mountain Systems; Broadleaf/Needleleaf/Mixed Forest | 81.00 |
| Cont. Asia; Tropical Mountain Systems; Burnt, Natural Forest Mosaic | 40.50 |
| Cont. Asia; Tropical Mountain Systems; Forest, Cropland Mosaic; Non-Frontier | 40.50 |
| Cont. Asia; Tropical Rainforest; Broadleaf/Needleleaf/Mixed Forest | 180.00 |
| Cont. Asia; Tropical Rainforest; Burnt, Natural Forest Mosaic | 90.00 |
| Cont. Asia; Tropical Rainforest; Forest, Cropland Mosaic; Non-Frontier | 90.00 |
| Cont. Asia; Tropical Shrub Cover | 39.00 |
| Cultivated and Managed Land | 5.00 |
| Grassland; Subtropical Dry Forest/Subtropical Steppe/Subtropical Desert | 4.00 |
| Grassland; Temperate Cont. Forest/Temperate Mountain Systems | 4.50 |
| Grassland; Temperate Steppe and Temperate Desert | 3.00 |
| Grassland; Tropical Dry Forest, Tropical Shrubland, Tropical Desert | 4.00 |
| Grassland; Tropical Mountain Systems/Subtropical Mountain Systems/Temperate Oceanic Forests | 6.00 |
| Grassland; Tropical Rainforest/Tropical Moist Deciduous Forest/Subtropical Humid Forest | 8.00 |
| Insular Asia; Subtropical Dry Forest; Broadleaf/Needleleaf/Mixed Forest | 96.00 |
| Insular Asia; Subtropical Dry Forest; Burnt, Natural Forest Mosaic | 48.00 |
| Insular Asia; Subtropical Dry Forest; Forest, Cropland Mosaic; Non-Frontier | 48.00 |
| Insular Asia; Subtropical Humid Forest; Broadleaf/Needleleaf/Mixed Forest | 169.00 |
| Insular Asia; Subtropical Humid Forest; Burnt, Natural Forest Mosaic | 84.50 |
| Insular Asia; Subtropical Humid Forest; Forest, Cropland Mosaic; Non-Frontier | 84.50 |
| Insular Asia; Subtropical Mountain Systems; Broadleaf/Needleleaf/Mixed Forest | 122.00 |
| Insular Asia; Subtropical Mountain Systems; Burnt, Natural Forest Mosaic | 61.00 |
| Insular Asia; Subtropical Mountain Systems; Forest, Cropland Mosaic; Non-Frontie | 61.00 |
| Insular Asia; Temperate Cont. Forest; Broadleaf Forest; Frontier | 69.00 |
| Insular Asia; Temperate Cont. Forest; Broadleaf/Needleleaf Forest; Non-Frontier | 14.00 |
| Insular Asia; Temperate Cont. Forest; Burnt, Natural Forest Mosaic; Frontier | 35.50 |
| Insular Asia; Temperate Cont. Forest; Burnt, Natural Forest Mosaic; Non-Frontier | 6.75 |
| Insular Asia; Temperate Cont. Forest; Forest, Cropland Mosaic; Non-Frontier | 6.75 |
| Insular Asia; Temperate Cont. Forest; Mixed Forest; Frontier | 71.00 |
| Insular Asia; Temperate Cont. Forest; Mixed Forest; Non-Frontier | 13.50 |
| Insular Asia; Temperate Cont. Forest; Needleleaf Forest; Frontier | 73.00 |
| Insular Asia; Temperate Mountain Systems; Broadleaf Forest; Frontier | 75.00 |
| Insular Asia; Temperate Mountain Systems; Broadleaf Forest; Non-Frontier | 58.00 |
| Insular Asia; Temperate Mountain Systems; Burnt, Natural Forest Mosaic; Frontier | 38.50 |
| Insular Asia; Temperate Mountain Systems; Burnt, Natural Forest Mosaic; Non-Frontier | 29.75 |
| Insular Asia; Temperate Mountain Systems; Forest, Cropland Mosaic; Non-Frontier | 29.75 |
| Insular Asia; Temperate Mountain Systems; Mixed Forest; Frontier | 77.00 |
| Insular Asia; Temperate Mountain Systems; Mixed Forest; Non-Frontier | 59.50 |
| Insular Asia; Temperate Mountain Systems; Needleleaf Forest; Frontier | 79.00 |
| Insular Asia; Temperate Mountain Systems; Needleleaf Forest; Non-Frontier | 61.00 |
| Insular Asia; Tropical Dry Forest; Broadleaf/Needleleaf/Mixed Forest | 96.00 |
| Insular Asia; Tropical Dry Forest; Burnt, Natural Forest Mosaic | 48.00 |
| Insular Asia; Tropical Dry Forest; Forest, Cropland Mosaic; Non-Frontier | 48.00 |
| Insular Asia; Tropical Moist Deciduous Forest; Broadleaf/Needleleaf/Mixed Forest | 169.00 |
| Insular Asia; Tropical Moist Deciduous Forest; Burnt, Natural Forest Mosaic | 84.50 |
| Insular Asia; Tropical Moist Deciduous Forest; Forest, Cropland Mosaic; Non-Frontier | 48.00 |
| Insular Asia; Tropical Mountain Systems; Broadleaf/Needleleaf/Mixed Forest | 122.00 |
| Insular Asia; Tropical Mountain Systems; Burnt, Natural Forest Mosaic | 61.00 |
| Insular Asia; Tropical Rainforest; Broadleaf/Needleleaf/Mixed Forest | 225.00 |
| Insular Asia; Tropical Rainforest; Burnt, Natural Forest Mosaic | 112.50 |
| Insular Asia; Tropical Rainforest; Forest, Cropland Mosaic; Non-Frontier | 61.00 |
| Sparse Grassland, Grassland Mosaic; Subtrop. Desert/Subtrop. Dry Forest/Subtrop. Steppe | 2.00 |
| Sparse Grassland, Grassland Mosaic; Subtropical Humid Forest | 4.00 |
| Sparse Grassland, Grassland Mosaic; Subtropical Mountain Systems | 3.00 |
| Sparse Grassland, Grassland Mosaic; Temp. Cont. Forest/Temp. Mountain Systems | 2.25 |
| Sparse Grassland, Grassland Mosaic; Temperate Oceanic Forests | 3.00 |
| Sparse Grassland, Grassland Mosaic; Temperate Steppe and Temperate Desert | 1.50 |
| Sparse Grassland, Grassland Mosaic; Tropical Dry Forest/ Tropical Shrubland | 2.00 |
| Sparse Grassland, Grassland Mosaic; Tropical Moist Deciduous Forest | 4.00 |
| Sparse Grassland, Grassland Mosaic; Tropical Mountain Systems | 3.00 |
| Sparse Grassland, Grassland Mosaic; Tropical Rainforest | 4.00 |

**Net greenhouse gas emissions:**

Table 3 presents the average emissions for each country in the HCT database. The methodology to estimate CO_2_ emissions is presented in the main text.

**Table 3.** CO_2_ emissions in metric tons per megawatt hour

| Country | CO_2_ emissions in metric tons  per megawatt hour |
| --- | --- |
| Argentina | 0.411100622 |
| Bhutan | 0.004000000 |
| Bolivia | 0.299750821 |
| Brazil | 0.107398560 |
| Burkina Faso | 0.682264151 |
| Burundi | 0.004000000 |
| Cambodia | 0.438418029 |
| Canada | 0.185975674 |
| Central African Republic | 0.096888888 |
| Chile | 0.474958610 |
| China | 0.727774884 |
| Colombia | 0.117727459 |
| Costa Rica | 0.088995023 |
| Democratic Republic of Congo | 0.005088992 |
| Ecuador | 0.309818666 |
| Ethiopia | 0.007562326 |
| Ghana | 0.235533002 |
| Guatemala | 0.371560226 |
| Guinea | 0.341777789 |
| Guyana | 0.840000000 |
| Honduras | 0.450380958 |
| India | 0.764062770 |
| Kenya | 0.398778607 |
| Laos | 0.091588354 |
| Lesotho | 0.004000000 |
| Mali | 0.194376245 |
| Mexico | 0.510174537 |
| Mozambique | 0.004855303 |
| Nepal | 0.346622942 |
| Nicaragua | 0.570905976 |
| Pakistan | 0.403088653 |
| Panama | 0.359598829 |
| Peru | 0.234992318 |
| Rwanda | 0.474250007 |
| Senegal | 0.773012320 |
| Sudan | 0.004000000 |
| Tanzania | 0.461373609 |
| Thailand | 0.564293875 |
| Uganda | 0.138190760 |
| United States | 0.530032888 |
| Venezuela | 0.197971092 |
| Vietnam | 0.419599695 |
| Zambia | 0.005970149 |
| Zimbabwe | 0.091699655 |

1. **Output interface**

Figure 6 presents the output table, which summarizes the main findings. Additionally to the results table, HCT also presents two graphics: the cumulative net present value and the greenhouse gas emissions. To keep the description brief, we present the later. Figure 7 shows that amount of CO_2_-equivalent, both gross and net, emitted by the hydropower project over its life-cycle.


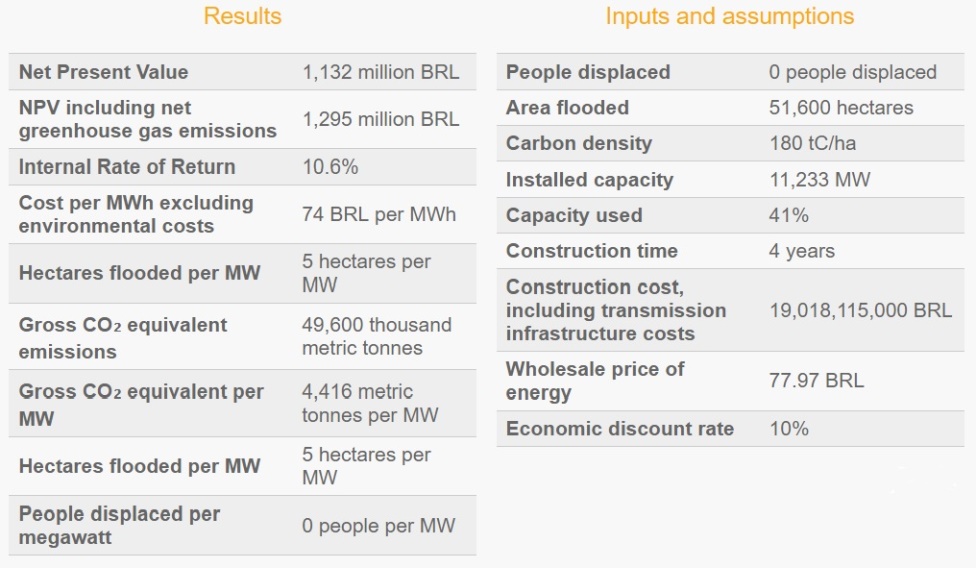


Fig 6. The HydroCalculator Tool's results for Belo Monte.


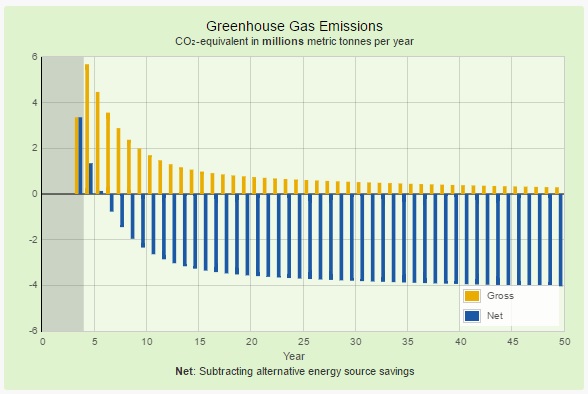


Fig 7. Greenhouse Gas Emissions for Belo Monte.

**Bibliography**

Abril G, Guérin F, Richard S, Delmas R, Galy-Lacaux C, Gosse P, Tremblay A, Varfalvy L, Dos Santos M A and Matvienko B 2005. Carbon dioxide and methane emissions and the carbon budget of a 10-year old tropical reservoir (Petit Saut, French Guiana). *Global Biogeochemical Cycles* **19** 1 - 16.

De Lima, I B T, Bambace L A W, and Ramos F M 2007. GHG life cycle analysis and novel opportunities arising from emerging technologies developed for tropical dams. Workshop on *the Greenhouse Gas Status of Freshwater Reservoirs*.

Fearnside, P M 2015. Emissions from tropical hydropower and the IPCC. *Environmental Science & Policy* **50** 225 - 239.

IPCC 2011. Special Report on Renewable Energy Sources and Climate Change Mitigation*.* Prepared by Working Group III of the Intergovernmental Panel on Climate Change: Cambridge University Press, Cambridge, UK and New York, NY.

IPCC 2013. Climate Change 2013: The Physical Science Basis. Contribution of Working Group I to the Fifth Assessment Report of the Intergovernmental Panel on Climate*.* Cambridge and New York: Cambridge University Press, 2013.

1. 1160 G Street, Suite A-1. Arcata, CA 95521 [↑](#footnote-ref-1)
